# Effects of Multiple-Hole Baffle Arrangements on Flow Fields in a Five-Strand Asymmetric Tundish

**DOI:** 10.3390/ma13225129

**Published:** 2020-11-13

**Authors:** Binglong Zhang, Fuhai Liu, Rong Zhu, Jinfeng Zhu

**Affiliations:** 1 BIOSCOPE Group, School of Metallurgical and Ecological Engineering, University of Science and Technology Beijing, Beijing 100083, China; B20160108@xs.ustb.edu.cn (B.Z.); zhurong@ustb.edu.cn (R.Z.); 2 National Center for Materials Service Safety, University of Science and Technology Beijing, Beijing 100083, China; 3 Tangshan Xinbaotai Steel Co., Ltd., Tangshan 064000, China; fanman2006@126.com

**Keywords:** tundish, diversion hole, water experiment, numerical simulation

## Abstract

This paper reports on the re-engineering of standard five-strand tundish designs into a five-strand asymmetric tundish, which resulted in a non-uniform rate and bias for each strand. We sought to improve the casting conditions by optimizing the liquid steel flow-field in the tundish. Both a water modelling experiment and a numerical simulation were performed to analyze the flow-field according to various diversion hole diameters and injection angles. The results showed that the average residence time decreased as the diameter of the diversion holes increased. As the injection angle was increased, the average residence time initially decreased and then increased. The liquid steel from the ladle shroud rapidly extended to the #2 and #3 strands in the original tundish, which reduced the likelihood of inclusion collision and coalescence.

## 1. Introduction

Tundishes are widely used to supply and distribute liquid steel into molds during continuous casting [1,2,3]. The different distances between the ladle shroud and each strand means that weirs, turbo-stoppers, and diversion holes with different injection angles have to be applied in multi-strand tundishes to provide effective control during industrial production processes [4,5]. Such control is, in most cases, focused on the steel cleanliness, by removing more inclusions from the liquid steel through prolonging its average residence time and reducing variations in the flow rate for each strand. In recent research, it has become standard practice to use both water experiments and numerical simulations to investigate the flow-field distribution in tundishes [6,7].

Numerous studies have been undertaken to attempt to establish the most effective tundish structure for managing the flow-field of liquid steel [8,9,10]. Chatterjee et al. [11], for instance, investigated the transient steel quality under non-isothermal conditions in a multi-strand billet caster tundish by water experiments and numerical simulations. Analysis of the results reported the effect of the non-isothermal condition on the temperature distribution and inclusion trajectories in the tundish. 

Chattopadhyay et al. [12] studied the effect of a dam and pad structure on the flow-field in a four-strand tundish through a combination of numerical simulations and experimental measurements and proposed an appropriate internal configuration on the basis of their results. Huang et al. [13] used digital particle image velocimetry technology in a synthetic hydraulic model to establish that turbulent flow is transient and random and that the characteristics of vortex flow are not center symmetrical. He et al. [14] built a simulation model to investigate the effect of casting speed on the flow pattern of a two-strand tundish and found that increasing the far-strand’s casting speed could achieve a better flow characteristic, comparing with increasing the casting speed of each strand simultaneously.

However, to date, little attention has been devoted to optimizing the structure of multi-strand asymmetrical tundishes. Sarkar et al. [15] analyzed the complex fluid flow and thermal transport in a five-strand asymmetric tundish through several numerical experiments and revealed that the maximum temperature drop was at the first run and became nearly identical at the end. Ramirez et al. [16] selected a multiphase model (Volume of Fluid) with a User Defined Function (UDF) to research the flow field and inclusion removal in a five-strand asymmetric tundish and proposed the difference between the UDF and the trap boundary condition in the removal of non-metallic inclusions with various diameters.

Despite this lack of prior research, the Tangshan Xinbaotai Steel company in China needed to improve the production capacity of its casting. A five-strand tundish was required but, after re-engineering the design, there was still not enough room for a standard five-strand tundish, so an asymmetrical tundish had to be built instead. This resulted in the generation of a non-uniform rate and bias for each strand. Therefore, the inclusion removal performance for each strand was different, which affected the quality stability for the billet. To solve this problem, research was undertaken to arrive at an optimal tundish design that could ensure the best metallurgical quality possible.

To conduct the research, both water and numerical modelling were undertaken to investigate the field-flow characteristics of an asymmetrical five-strand tundish. The residence time and standard deviation values were measured in the water modelling experiment, using different diameters and injection angles for the diversion holes. The velocity, temperature, and inclusion removal rate distributions were examined using the simulation. On the basis of the results, a proposed optimum diversion hole arrangement was tested in a five-strand tundish.

## 2. Water Model Experiment

### 2.1. Experimental Principle

On the basis of previous research, a water modelling model was built according to the similarity principle [17,18]. The basic conditions of the liquid flow similarity between the model and the prototype were geometric and dynamic similarity. The parameters used in the model were calculated using the Froude similarity criterion, which can be expressed as follows:

(1) $$Fr_{m}=Fr_{p}.$$

From which,

(2) $$\frac{V_{m}^{2}}{gL_{m}}=\frac{V_{p}^{2}}{gL_{p}}$$

where the subscript *m* and *p* represent the water model and prototype of the tundish, respectively; and *V*, *g*, and *L* are the flow velocity, gravity, and characteristic length, respectively.

**Figure 1:**
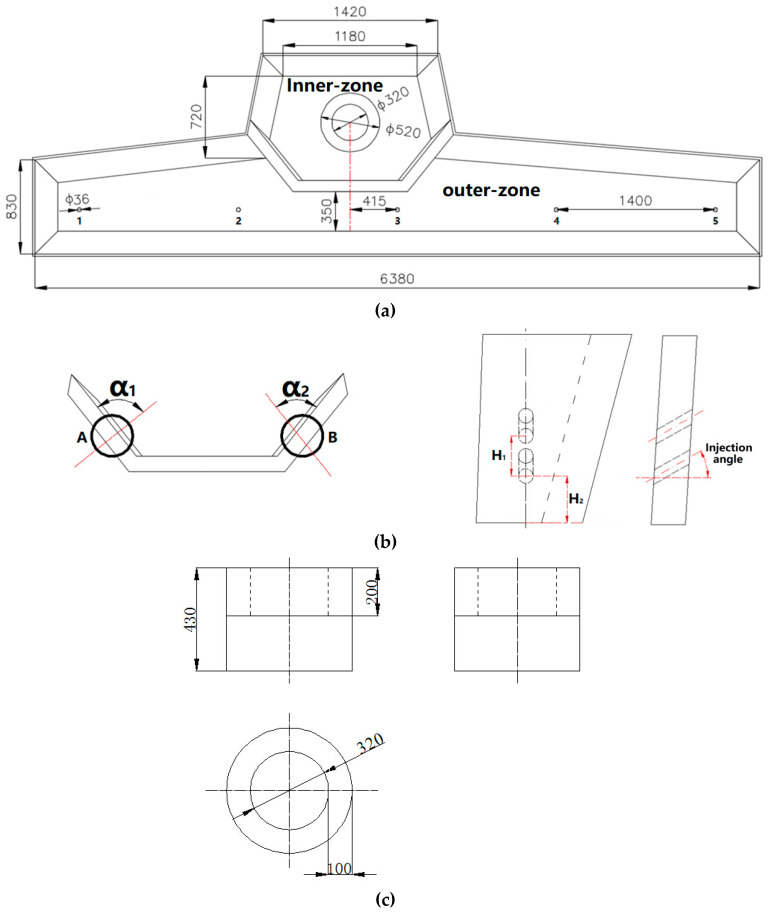
(**a**) Parameters for the tundish prototype. (**b**) Sketch view of the diversion wall. (**c**) Sketch view of the turbulence inhibitor (units in millimeters).

The parameters for the prototype and the model are shown in Figure 1 and Table 1. From Figure 1a,b, the designed diameter for the diversion hole at each side of the diversion wall was different. The diversion holes to the left-hand and the right-hand sides are labelled henceforth as diversion holes A and B, respectively. Similarly, the injection angles for diversion holes A and B will be termed α_1_ and α_2_, respectively. There was no diversion wall or turbulence suppressor in the original tundish.

### 2.2. Experiment Instruments

**Figure 2:**
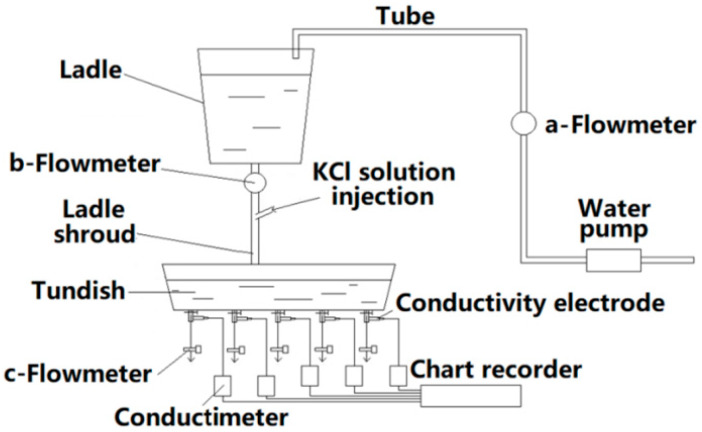
Experiment apparatus.

The modelling apparatus (see Figure 2) included a tundish, a ladle, a flowmeter, a water pump, a ladle shroud, a conductivity electrode, a conductimeter, and a chart recorder. Tap water was used in the experiment in place of liquid steel. The tundish and ladle were made of plexiglass with a ratio of model to tundish of 1:3. The flowmeters labeled a, b, and c were used to control the water depth in the ladle and the inlet flow rate for the tundish, respectively. 

To obtain a residence time distribution (RTD) curve, five conductivity electrodes were installed separately at the tundish outlets. When a steady-state flow condition was attained, a 200 mL KCl saturated solution was used as a tracer to measure the electronic conductivity of the water in the different outlets.

Figure 1 shows the shape of the five-strand tundish, with each strand being assigned a number. The residence times obtained for the #1, #2, #3, #4, and #5 strands are designated as *RT*_1_, *RT*_2_, *RT*_3_, *RT*_4_, and *RT*_5_, respectively. Then, the residence time (RT)—which is henceforth called R-time—for a certain diversion hole arrangement, was calculated as an arithmetical mean using Equation (3):

(3) $$RT=\frac{RT_{1}+RT_{2}+RT_{3}+RT_{4}+RT_{5}}{5}.$$

The standard deviation of the residence time (*RT*_s_) was calculated using the following Equation (4):

(4) $$RT_{S}=\sqrt{\frac{(RT_{1}-RT{)}^{2}+(RT_{2}-RT{)}^{2}+(RT_{3}-RT{)}^{2}+(RT_{4}-RT{)}^{2}+(RT_{5}-RT{)}^{2}}{5}}.$$

## 3. Numerical Simulation

### 3.1. Governing Equations

The simulation model of multiphase behavior under turbulent conditions was based on the Navier–Stokes equation. [19] The basic mathematical models describing the continuity, momentum, and energy conservation were based on the following Equations (5)–(7):

Continuity:

(5) $$\frac{\partial \rho }{\partial t}+\rho \left(\nabla u\right)=0,$$

Momentum:

(6) $$\rho \frac{\partial (u)}{\partial t}+\rho \left[u\cdot \nabla \right]u=-\nabla P+\mu_{eff}\nabla^{2}u+\rho g,$$

and energy:

(7) $$\frac{\partial }{\partial t}(\rho T)+u\cdot \nabla [\rho T]=\nabla \cdot [(\frac{\kappa }{C_{P}}+\frac{\mu_{t}}{\sigma_{h}})\nabla T],$$

where *ρ*, *t*, and *u* are the time, fluid density, and velocity, respectively; the *P*, *T*, *C_p_*, and g are the pressure, temperature, specific heat capacity, and gravity acceleration, respectively; $\vec{J_{i}}$ and *h_i_* are the diffusion flux and enthalpy of species *i*. *μ_eff_* is the sum of the molecular viscosity, *κ* is the conductivity, and *μ_t_* is the turbulent viscosity. *σ_h_* is a constant with a value of 0.9 [15].

In this research, the gas phase is defined as an ideal gas, and the liquid phase (liquid slag and molten steel) is defined as an incompressible flow. Hence, Equations (8) and (9) represent the sensible enthalpy (*h*) for the gas phase and liquid phase, respectively.

(8) $$h=\sum_{i}Y_{i}h_{i}$$

(9) $$h=\sum_{i}Y_{i}h_{i}+\frac{P}{\rho }$$

where *Y_i_* is the mass fraction of species *i*.

A standard *k*-*ε* model was used to predict the Reynolds stress tensor, which completed the RANS (Reynolds average Navier-Stockes) equations for the simulation process [5,16,18]. Equations (10) and (11) represent the turbulent kinetic energy (*k*) and its dissipation rate (*ε*), based on the transport equation:

(10) $$\frac{\partial (\rho k)}{\partial t}+\frac{\partial (\rho ku_{i})}{\partial x_{i}}=\frac{\partial }{\partial x_{j}}[(\mu +\frac{\mu_{t}}{\sigma_{\kappa }})\frac{\partial k}{\partial x_{j}}]+G_{k}+G_{b}-\rho \varepsilon$$

(11) $$\frac{\partial (\rho \varepsilon )}{\partial t}+\frac{\partial (\rho \varepsilon u_{i})}{\partial x_{i}}=\frac{\partial }{\partial x_{j}}\left((\mu +\frac{\mu_{t}}{\sigma_{\varepsilon }})\frac{\partial \varepsilon }{\partial x_{j}}\right)+C_{1\varepsilon }\frac{\varepsilon }{\kappa }(G_{k}+C_{3\varepsilon }G_{b})-\rho C_{2\varepsilon }\frac{\varepsilon^{2}}{\kappa }$$

where *C*_1*ε*_, *C*_2*ε*_, *σ_k_*, and *σ_ε_* are constants for the *k*-*ε* model, with values of 1.38, 1.92, 1.0, and 1.3, respectively [19,20]. For buoyant shear layers for which the main flow direction is aligned with the direction of gravity, *C*_3*ε*_ will become 1. For buoyant shear layers that are perpendicular to the gravitational vector, *C*_3*ε*_ will become 0 [21]. *G_k_* and *G_b_* represent the generation of turbulent kinetic energy in relation to the mean velocity gradient and buoyancy [18].

In this research, the volume of fluid (VOF) model was used to investigate the characteristics of the surface motion for the multi-phase. [19] For the VOF model, two or more phases were not interpenetrating, and each phase had its own volume fraction (*α*). In each cell, the sum of all phases was equal to unity:

(12) $$\sum_{i=1}^{n}\alpha_{i}=1.$$

The variables in the VOF model were achieved by the volume average method using the volume fraction. For the gas–slag–steel system in the tundish, the effective viscosity (*μ*) can be calculated by:

(13) $$\mu =\alpha_{gas}\mu_{gas}+\alpha_{slag}\mu_{slag}+\alpha_{steel}\mu_{steel}.$$

The behavior of the inclusion in the molten steel was simulated using a Lagrangian discrete phase model in the Eulerian–Eulerian reference frame. The local particle average velocity components were obtained by solving the following inclusion momentum Equations (14)–(16):

(14) ∂up∂t=18μCDRe24ρpdp2(u−up)+g(ρp−ρ)ρp+0.5ρρp∂(u−up)∂t

where *ρ_p_* and *d_p_* are the density and diameter of an inclusion particle, respectively; *v*_*i*,*p*_ is the average velocity component for the inclusion particle in the *i*th directions; and Re and *C_D_* represent the Reynolds number and drag coefficient, which were defined as [19]:

(15) Re=ρdp(u−up)μ

(16) CD=a1+a2Re+a3Re2.

### 3.2. Simulation Details

**Figure 3:**
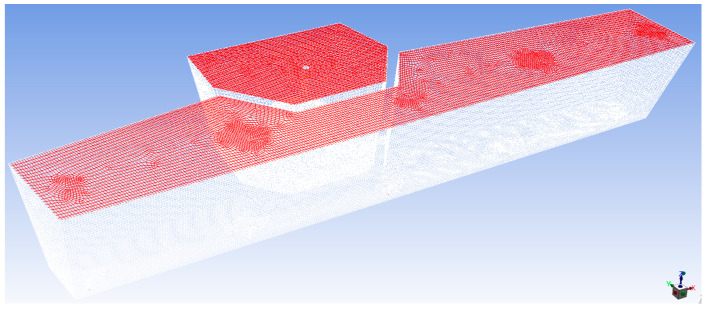
Mesh distribution of the five-strand tundish.

A three-dimensional simulation model for a full-size tundish with various diversion hole arrangements was developed to research the characteristics of the flow field. As shown in Figure 3, a tetrahedron and hexahedron were used to divide the calculation domain of the tundish with 1.44 million cells. In this research, the chemical reactions between the inclusion and the liquid steel were not considered. The pressure was set to a constant value (atmospheric) at the surface, which was equal to the atmospheric pressure, and the liquid slag and molten steel were treated as incompressible viscous fluids. The details of the thermo-physical properties of the three-phase material used in the model are presented in Table 2.

Alumina inclusions with an overall density of 3986.71 kg/m^3^ and consisting of a group of 10,000 particles with a size range of 10–100 μm, were used to represent the spherical inclusions present in the steel. A User Defined Function (UDF) was programmed, and used as one kind of trap boundary condition to simulate the attachment of inclusions to the upper slag layer [16,21]. This trap process for the inclusions tracked the particle phase in a certain cell, which was close to the surface between the liquid slag and molten bath. When the particle reached the surface, the cell including this particle was marked first, and then the velocity of this particle (*u_p_*) was computed. If *u_p_* was lesser than the critical velocity [22], this particle would be trapped and removed. Otherwise, this particle returned to the molten steel, and continued to move until it approached the surface again. 

The inclusions were injected through the shroud, and the number coming out from each strand was calculated using a transient model. The inclusion trajectories and inclusion removal rate (IRR) were calculated under non-isothermal condition. The IRR can be calculated using the following Equation (17):

(17) $$\mathrm{IRR}=\frac{\mathrm{Number}\;\mathrm{of}\;\mathrm{inclusions}\;\mathrm{of}\;a\;\mathrm{particular}\;\mathrm{size}\;\mathrm{range}\;\mathrm{resided}\;\mathrm{in}\;\mathrm{the}\;\mathrm{tundish}}{\mathrm{Number}\;\mathrm{of}\;\mathrm{inclusions}\;\mathrm{of}\;a\;\mathrm{particular}\;\mathrm{size}\;\mathrm{range}\;\mathrm{injected}\;\mathrm{into}\;\mathrm{the}\;\mathrm{tundish}}.$$

‘Reflect’ boundary conditions were used for the other tundish walls, and a standard wall function was applied to all of the tundish walls. This indicates the adhesion of non-metallic inclusions to the tundish wall was ignored. The regions inside and outside the diversion wall were defined here as the ‘inner-zone’ and ‘outer-zone’, respectively. 

The tundish inlet and strand outlet were defined as the velocity-inlet and outflow conditions. Based on the casting speed, the inlet velocity of steel from the ladle was 1.93 m/s, and its inlet temperature was 1843 K. As the literature reported [5,15,16], the heat transfer coefficient for the dams, top surface, bottom wall, side walls, and front and back walls were established to be 1.75, 15, 1.4, 3.8, 3.2, and 3.2 k W/m^2^, respectively.

A pressure-based solver with an unsteady-state model was adopted to simulate the flow-field of the liquid steel. The pressure–velocity coupling was solved using an explicit coupling scheme and second order spatial discretization was used to solve the pressure. Other variables, including the energy, turbulent kinetic energy, and dissipation rate, were calculated using a Second Scheme Solution. The convergence for the numerical model was determined using an energy residual of <10^−6^, with residuals for the other variables being set at <10^−5^. The initial time step size was 10^−4^ s to promote the fast convergence of all variables, and then an adaptive method was chosen for the time steps with the global Courant number being 1. The ending calculation time of the simulation model was set to 1600 s for each case.

### 3.3. Validation of the Simulation Model

**Figure 4:**
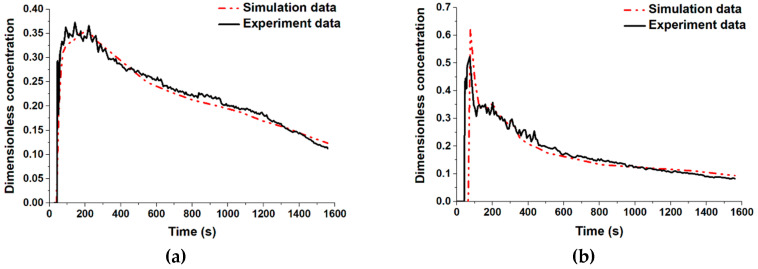
Comparison between the residence time distribution (RTD) curves for the experiment and the simulation. (**a**) Strand #2 and (**b**) Strand #4.

To ensure the accuracy of the simulation model, we investigated the RTD curve obtained by the water experiment and the numerical simulation. As the water experiment was an isothermal model, the heat loss of the molten steel was also not considered in the simulation model at this section. That means that all the tundish walls were defined as a temperature thermal conditions of 1843 K, and the back flow temperature of the outlet was also 1843 K. Figure 4 presents the respective RTD curves achieved by both the water experiment and the numerical simulation at strands #2 and #5. The dimensionless concentration value was unchanged at first after injecting the KCl saturated solution as it took some time for KCl to reach the exit of the strand. Then, the dimensionless concentration value rapidly increased to the maximum, when there was a large amount of the KCl passing though the strand. At last, the dimensionless concentration gradually decreased. The RTD curve for the numerical simulation was in good agreement with the water experiments. The validation shows that the results of numerical simulation were reliable, and the subsequent research for the numerical simulation section still took the heat transfer into consideration.

## 4. Results and Discussions 

### 4.1. Average Residence Time Distributions

**Figure 5:**
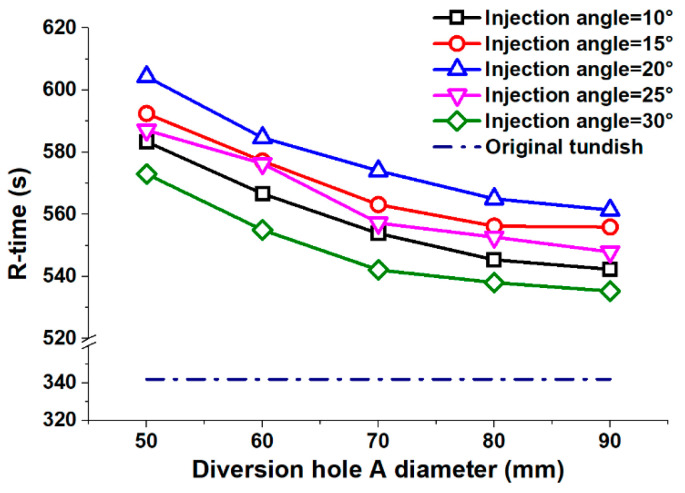
Average residence time distribution.

The residence time represents how long the liquid steel stays in the tundish after it has been injected from the ladle shroud. The longer the residence timer, the more inclusions can be removed with the slag. Figure 5 shows the profiles for the mean residence time according to different diversion hole arrangements. In a tundish with a division, there are two diversion holes, A and B, as presented in Figure 1b. In this research, the diameter variation between A and B was a certain value (15 mm) for various diversion hole arrangements, and the diameter of B diversion hole was the larger one. Thus, we used only the diameter of the A diversion hole to present the diameters of both the A and B diversion holes. For example, a 50 mm diversion hole arrangement represented the A and B hole diameter at 50 and 65 mm, respectively. The 70 mm–20° arrangement presented an arrangement with the A hole diameter of 70 mm, B hole diameter of 85 mm, and injection angle of 20°. The tundish without any structure of the diversion wall and the turbulence inhibitor was defined as the original tundish.

The R-time for the original tundish was 342 s, which was much shorter than it was for the tundish with a diversion hole arrangement. In relation to the original tundish, all the diversion hole arrangements proposed here improved the average residence time, leading to a better inclusion removal rate.

The test results showed that the R-time reduced as the diameter of the diversion hole increased. Thereby, the 90 mm diversion hole arrangement achieved the shortest average R-time of 548 s. The 50 mm diversion hole arrangement had the longest average R-time of 588 s. Figure 5 shows that the linear regressions slopes were −1.61, −1.39, −0.66, and −0.29 for the A diversion hole diameters of 50 mm–60°, 60 mm–70°, 70 mm–80°, and 80 mm–90°, respectively. This indicates that the downward trend for the average residence time decelerated as the A diversion hole diameter increased. 

The diversion hole and injection angle transmit the momentum of the liquid steel both radially and axially. A small injection angle increases the radial component of the momentum and decreases the axial element. A large injection angle has the opposite effect. The experimental results showed that the average residence time initially increased and then decreased as the injection angle increased. A 30° injection angle led to the shortest average R-time of 549 s. A 20° injection angle produced the longest average R-time of 578 s. On this basis, a 20° injection angle was best suited to prolonging the average residence time and improving the inclusion removal rate. Overall, a 50 mm−20° diversion hole arrangement was capable of generating the longest average R-time. This arrangement was found to produce a 604 s average residence time, which was 7.2% better than any of the other diversion hole arrangements.

### 4.2. Residence Time Standard Deviation Distributions

**Figure 6:**
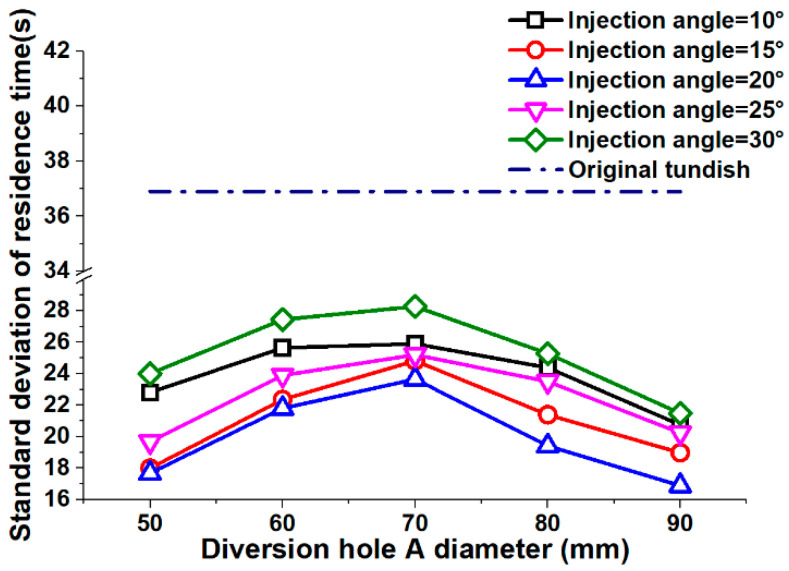
Residence time standard deviation distribution.

Bias can also have an effect on the residence time for the different strands, affecting, in turn, the control process for continuous casting. To this end, the standard deviation of the residence time was also calculated to analyze the variation for each stand. A lower standard deviation indicates that the data was closer to the mean value, which means that the bias phenomenon was suppressed. The standard deviation of the residence time for the original tundish was 36.9 s, which is significantly more than it was for the tundishes with diversion hole arrangements (see Figure 6). Thus, we conclude from this that a diversion hole arrangement will reduce the average standard deviation and diminish any possible bias effect.

Figure 6 depicts the residence time standard deviation (RT_s_) distribution according to different diversion hole arrangements. As the injection angle increases, the RT_s_ first decreases and then increases. The results showed that a 30° injection angle produced the biggest largest RT_s_ at 25.3 s, while a 20° injection angle delivered the smallest average RT_s_ at 19.9 s. In an opposite trend to the results for the injection angle, the standard deviation for the diversion hole diameter first increases and then decreases as the diameter increases. Thus, in the tests, a 70 mm diameter hole arrangement produced the largest average standard deviation at 25.6 s, and the 90 mm diameter arrangement produced the smallest average standard deviation at 16.7 s. 

Although the 90 mm−20° diversion hole arrangement delivered the smallest standard deviation at 16.7 s, the 50 mm−20° arrangement had the second smallest standard deviation at 20.4 s. When compared to the 90 mm−20° arrangement, the standard deviation was only 4.0% higher for the 50 mm−20° arrangement and there were 12 kinds of diversion hole arrangements that obtained a longer average R-time than the 90 mm−20° arrangement. In view of this, the 50 mm−20° arrangement was selected as the best diversion hole arrangement for removing inclusions and suppressing bias phenomena for a five-strand asymmetric tundish. However, to check the validity of this conclusion, a numerical simulation was also conducted to analyze the flow-field in the tundish.

### 4.3. Dead Volume Distributions

**Figure 7:**
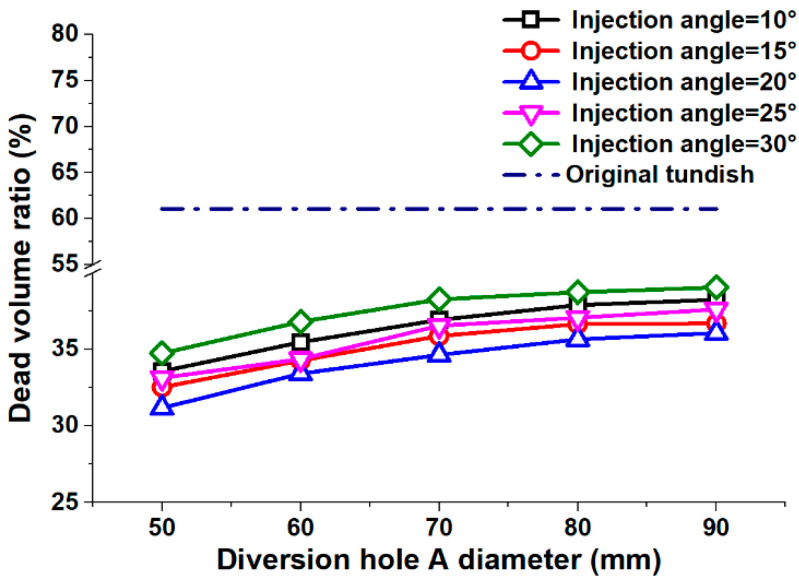
Dead volume ratio distribution.

For the tundish, a lower dead volume ratio indicates a better flow characteristic. The dead volume ratio can be calculated with Equation (18):

(18) $$V_{d}=1-(\frac{t_{a}}{t_{theory}})=1-(\frac{t_{a}}{T_{V}/F_{inlet}})$$

where *V_d_*, *T_V_*, and *F_inlet_* are the dead volume ratio, steel volume in the tundish, and steel flow rate, respectively; t_a_ and *t_theory_* are the average residence time and theoretical residence time, respectively. Figure 7 shows the profiles for the dead volume ratio according to different diversion hole arrangements.

The dead volume ratio of the original tundish was 61.0%, which was much bigger than it was for the tundish with a diversion hole arrangement at the tested conditions. Therefore, the diversion hole arrangement designed in this research was able to suppress the dead volume zone in the tundish, resulting in a greater flow field.

The simulation results showed that the dead volume ratio increased with a larger diameter of the diversion hole. Therefore, the 90 mm diversion hole arrangement achieved the largest average dead volume ratio of 37.5%. The 50 mm diversion hole arrangement had the smallest average dead volume ratio of 33.0%. Figure 7 shows that the linear regressions slopes were 0.18, 0.16, 0.08, and 0.03 for the A diversion hole diameters of 50 mm–60°, 60 mm–70°, 70 mm–80°, and 80 mm–90°, respectively. This indicates that the upward trend for the average dead volume initially increased and then reduced as the A diversion hole diameter increased.

The average residence time first increased and then decreased with the increasing injection angle. A 30° injection angle formed the largest average dead volume ratio of 37.5%. A 20° injection angle achieved the smallest average dead volume ratio of 37.5%. That indicates that a 20° injection angle arrangement was effective to suppress the dead volume and improve the flow field in the tundish. Based on the results, a 50 mm−20° diversion hole arrangement was best suited to form the smallest average dead volume ratio of 31.2%, which was 12.9% smaller than any of the other diversion hole arrangements.

### 4.4. Velocity and Vector Distributions

**Figure 8:**
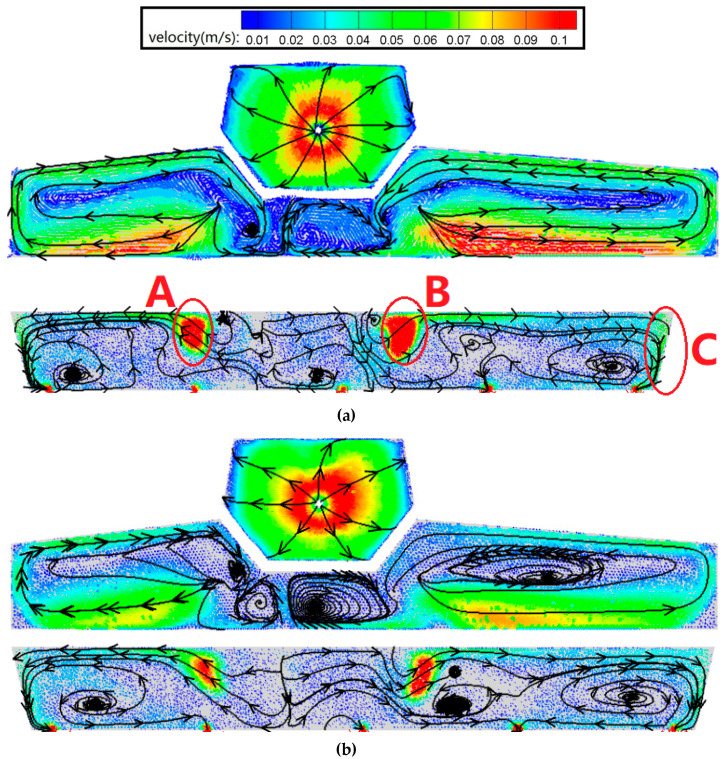

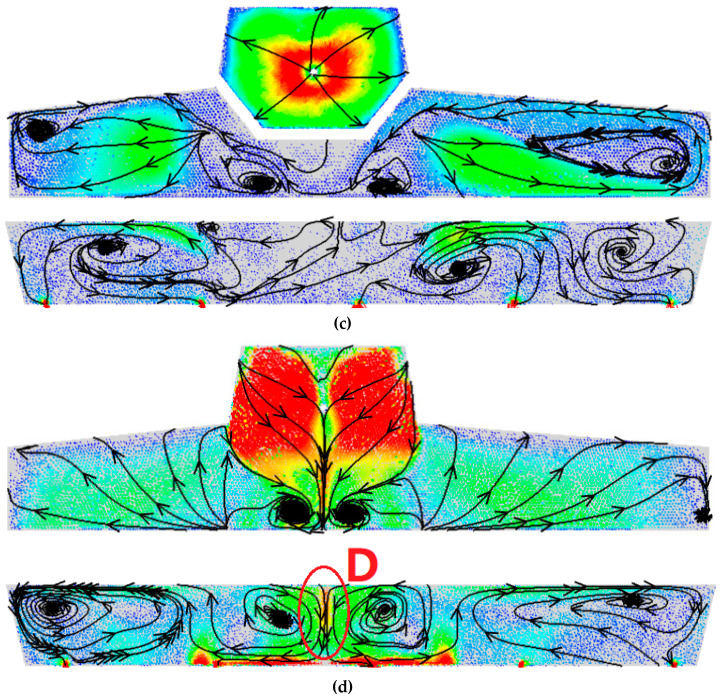
The velocity and vector distributions in the tundish for a longitudinal cross-section according to different internal structures. (**a**) The 50 mm−20° arrangement; (**b**) 70 mm−20° arrangement; (**c**) 90 mm−20° arrangement; and (**d**) original arrangement.

The velocity and vector distribution in the tundish according to different diversion hole arrangements is shown in Figure 8. The velocity of the liquid steel was controlled by passing it though a diversion hole, with the shift in velocity gradient enabling some of the kinetic energy to be absorbed by the lower-velocity liquid steel.

In the case of a diversion hole arrangement, the pressure difference between the inner and outer zones in a tundish has a set value, with the initial velocity of the liquid steel in the A and B diversion holes being essentially the same. Therefore, if diversion hole A has a smaller diameter, the area (at a velocity of 0.05 m/s) of region A will be smaller than region B. As the diameter of diversion holes A and B increases, the inhibiting effect of the diversion wall is suppressed, which reduces the pressure difference between the inner and outer zones. Thus, the bigger the diameter of the diversion holes, the lower the initial velocity of the liquid steel. As a result, as the diameter of the A-diversion hole increased from 50 to 90 mm, the area of the high-velocity zone continued to reduce while the inlet flow-rate of the liquid steel from the ladle remained constant. 

There was also a medium-velocity zone (region C), which was formed by the whirl of the liquid steel extending from the surface to the side-wall. In the original tundish, the speed of the liquid steel at the bottom of the wall below the ladle shroud (region D) was much higher than it was in a tundish with a diversion hole arrangement. As a result, the liquid steel traversed two of the strands (#2 and #3), creating a bias phenomenon and leading to a lower residence time (see Figure 8b). 

Slag entrapment is one of the reasons why the inclusion in liquid steel may increase, which is inimical to the quality of the steel. Slag entrapment arises due to surface fluctuations between the liquid steel and the slag. It is important, in that case, to measure the average axial velocity at the surface of the tundish to evaluate the surface fluctuation. A transverse plane (plane A) was taken 5 mm below the surface of the tundish surface because the adhesion boundary condition resulted in the surface-wall velocity being 0 m/s. The average axial velocity of the liquid steel at plane A using the 50 mm−20°, 60 mm−20°, 70 mm−20°, 80 mm−20°, 90 mm−20°, and 0 mm−0° arrangements was 9.7 × 10^−6^, 8.2 × 10^−6^, 11.4 × 10^−6^, 10.8 × 10^−6^, 17.8 × 10^−6^, and 74.7 × 10^−6^ m/s, respectively. The 50 mm−20° arrangement produced the second smallest average axial velocity, which was 56.1% lower than other kinds of arrangements. This indicates that a 50 mm−20° arrangement is not likely to result in slag entrapment.

### 4.5. Static Temperature Distributions

**Figure 9:**
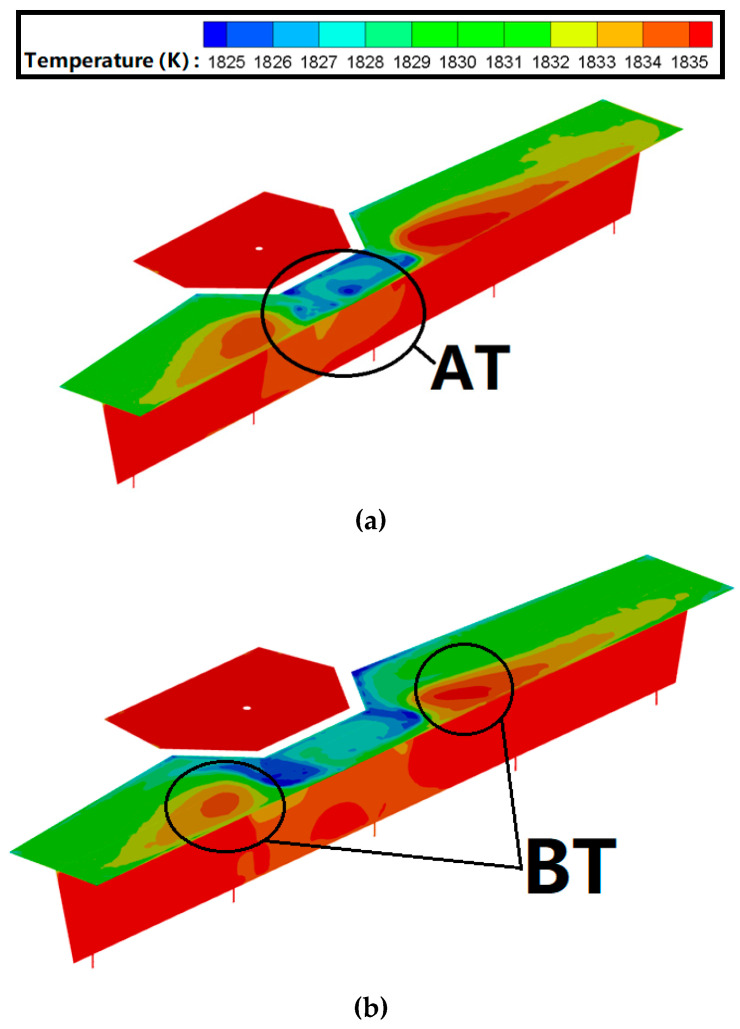

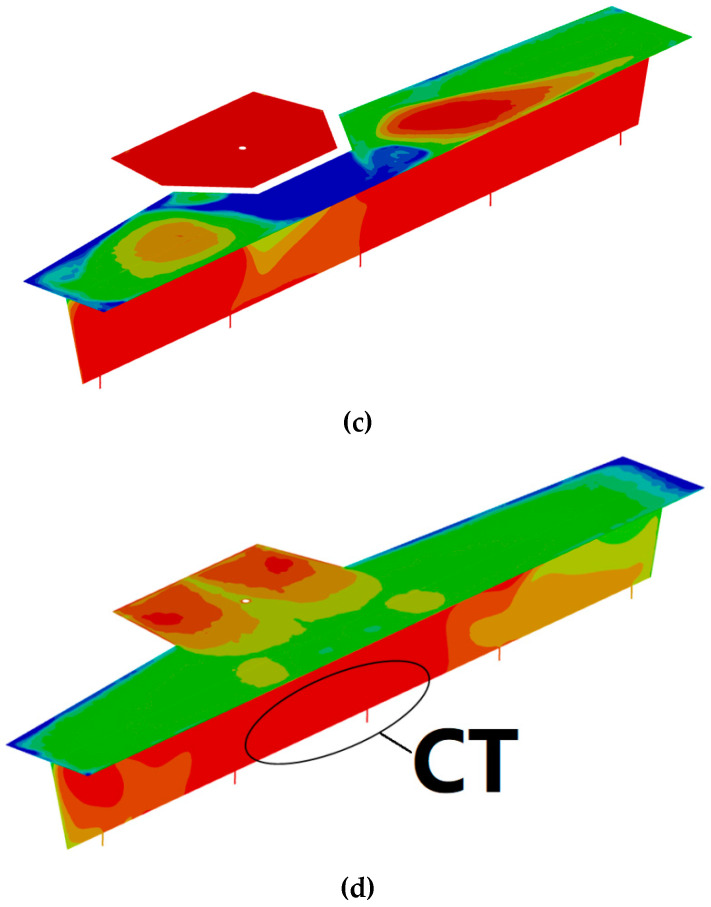
The static temperature distribution in a tundish with a longitudinal cross-section according to different internal structures. (**a**) The 50 mm−20° arrangement; (**b**) 70 mm−20° arrangement; (**c**) 90 mm−20° arrangement; and (**d**) original arrangement.

In this paper, static temperature is generally shortened to ‘temperature’. For a tundish using a diversion hole arrangement, there is a lower temperature zone (region AT) at the tundish surface near the diversion wall. The liquid steel extends from the surface to the side-wall after passing though the diversion hole, resulting in it losing more heat energy in the region AT and, thus, generating a lower temperature zone (see Figure 9). The region AT expands as the diameter of the diversion hole increases. At the same time, after the liquid steel passes through the diversion hole, it will quickly mix with the surface of molten bath, resulting in two higher temperature zones (region BT). In the original tundish, the liquid steel will not directly flow up to the surface of the molten bath without the diversion hole, resulting in the temperature of the liquid steel in the region BT being lower. 

Table 3 shows the average temperature distribution for molten steel. The results showed that the average temperature of the molten bath increased as the diameter of the diversion hole reduced. The average temperature in the original tundish was 1833.6 K, which was 1.8 K lower than the tundish using a diversion hole arrangement. As mentioned, the molten steel will quickly flow to the bottom of the tundish and then discharge from the #2 and #3 strand. Therefore, a higher temperature zone (region CT) was formed at the center of the tundish, which nears the #2 and #3 strands. This indicates that the high-temperature molten steel could not efficiently transfer its heat energy to the low-temperature molten steel at other regions of the tundish, resulting in the average temperature of molten bath being lower.

At the surface and the bottom of the molten steel, the average temperature in the tundish using a diversion hole arrangement was 1832.3 K and 1836.1 K, respectively; thus, there was a temperature variation of 3.8 K. The average temperature in the original tundish at the surface and the bottom of the molten steel was 1830.2 and 1834.7 K, respectively; thus, there was a temperature variation of 4.5 K. The diversion hole arrangement, therefore, reduced any loss or variation in the tundish temperature. The average temperature distribution of the molten steel surface and bottom both increased as the diameter of the diversion hole reduced, which was the same as the average temperature variation between the surface and the bottom of the molten steel.

### 4.6. Inclusion Removel Rate Distributions

**Figure 10:**
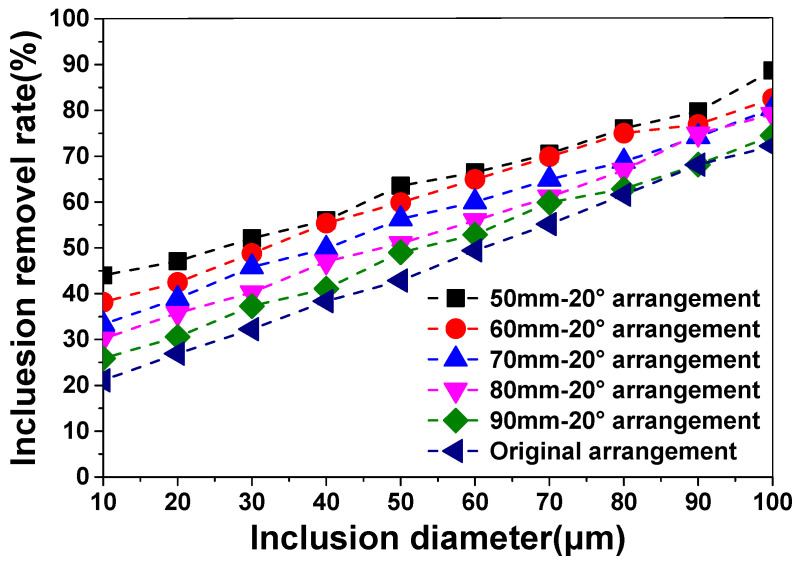
The inclusion removal rate (IRR) distribution for different inclusion sizes.

Figure 10 shows the IRR distribution for different inclusion sizes. The simulation results showed that the IRR increased as the diameter of the diversion hole reduced and decreased as the diameter of the inclusions reduced. The results showed that an original arrangement achieved the smallest average IRR of 46.8%, which was 10.7% lower than the overall IRR average for a tundish using a diversion hole arrangement. This indicates that the different-sized inclusions will be directed to the slag layer by the diversion wall and turbulence suppressor, resulting in a larger average IRR when using a diversion hole arrangement.

A 50 mm diversion hole arrangement produced the largest average IRR of 64.4%, which was 6.9% higher than the overall IRR average for tundishes using a diversion hole arrangement. At the tested condition, the 50 mm diversion hole arrangement achieved the largest average IRR for different-sized inclusions, and was more suitable for this five-strand asymmetric tundish, which is consistent with the conclusion of the water experiment.

## 5. Conclusions

This paper reported on the effects of different diversion hole diameters and injection angles on the flow-field in a five-strand asymmetric tundish. The results showed that the data obtained using the water experiment and the numerical simulation agreed well with actual industrial applications. The main conclusions can be summarized as follows:

1. The average residence time reduced as the diameter of the diversion hole increased. However, the average standard deviation for each strand first increased and then decreased with an increase in the diversion hole diameter. With regard to the injection angle, both the average residence time and standard deviation first decreased and then increased as the injection angle increased.
2. For the original tundish, the speed of the liquid steel at the bottom of the wall below the ladle shroud (region D) was much higher than it was in a tundish with a diversion hole arrangement. As a result, the liquid steel traversed two of the strands (#2 and #3), creating a bias phenomenon and leading to a lower residence time.
3. The average temperature of the molten bath increased as the diameter of the diversion hole reduced. The average temperature in the original tundish was 1833.6 K, which was 1.8 K lower than the tundish using a diversion hole arrangement.
4. The different-sized inclusion will be directed to the slag layer by the diversion wall and turbulence suppressor, resulting in a larger average IRR when using a diversion hole arrangement. As a result, the original tundish achieved the smallest average IRR of 46.8%, which was 10.7% lower than the overall IRR average for a tundish using a diversion hole arrangement.

## Figures and Tables

**Table 1:** Geometrical parameters for the prototype and model.

Parameter	Prototype	Model
Flow rate (m^3^/h)	16.5	1.058
Depth of the liquid steel (mm)	750	250
Ladle shroud diameter (mm)	55	18.3
Immersion depth of Ladle Shroud diameter (mm)	180	60
α_1_ and α_2_ (°)	15	15
Injection angle (°)	10, 15, 20, 25 and 30	10, 15, 20, 25 and 30
A diversion hole diameter (mm)	50, 60, 70, 80 and 90	16.7, 20.0, 23.3, 26.7 and 30.0
B diversion hole diameter (mm)	65, 75, 85, 95 and 105	21.7, 25.0, 28.3, 31.7 and 35.0
H_1_/H_2_ (mm)	150/200	50.0/66.7

**Table 2:** The thermo-physical properties of the three-phase material.

Material/Interphase	Density (kg/m^3^)	Viscosity (kg/m·s)	Cp (J/kg·K)	Thermal Conductivity (W/m·K)	Surface Tension (N/m)
Steel	7010	0.0061	755	41	—
Slag	2650	0.34	875	8.1	—
Air	1.225	1.789·10^−5^	1006	0.0242	—
Steel-slag	—	—	—	—	0.12
Air-steel	—	—	—	—	1.60
Air-slag	—	—	—	—	1.40

**Table 3:** The average temperature distribution for the molten steel.

Label		50 mm−20°	60 mm−20°	70 mm−20°	80 mm−20°	90 mm−20°	Original Tundish
**Average Temperature (K)**	Molten bath	1835.8	1835.7	1835.5	1835.2	1835.0	1833.6
	Surface of the molten steel	1833.1	1832.8	1832.5	1831.8	1831.5	1830.2
	Bottom of the molten steel	1836.4	1836.3	1836.1	1836.0	1835.8	1834.7
